# Supplementary material for: Learning curve and surgical outcome of robotic assisted colorectal surgery with ERAS program
Source: Sci Rep. 2022 Nov 29;12:20566. doi: 10.1038/s41598-022-24665-w (PMC9709162; doi:10.1038/s41598-022-24665-w)
Supplement: Supplementary file 1 — Supplementary Information 1. [file 41598_2022_24665_MOESM1_ESM.zip › LSC Raw data-Ñ[▒K/048/OP_ñw╝╨░O▒Kñσ.pdf]

正式摘要(電子簽章：000000177350978)

W55 / W55

臺中榮民總醫院

Operation Note

[redacted]

- 手術日期：2019/10/05
- 手術醫師：VS 蔣鋒帆，R 陳映菁，R 林陳彥，R 林楙時，R 鄭力云
- Anesthesia : GE
- Position : Supine
- Aseptic agents : Soap , Betadine Alcohol , Betadine Aqua

Diagnosis

- Pre-operative diagnosis : CA OF LOW RECTUM
- Post-operative diagnosis : Ditto., s/p laparoscopic TME+ loop ileostomy

Surgical Indication

- To remove lesion for pathologic proof and staging

Operation

- 1. Laparoscopic TME+ loop ileostomy
  2. Loop ileostomy at RLQ
  3. IMA ligation
  4. Cut margin: proximal 10 cm, distal 1 cm
  5. Take down of splenic flexure: (-)
  6. A Fr.12 Penrose drain placed in the pelvis

Finding

- Operative findings:
  1. A 2.5*2.5cm? Bormann's type III? tumor at lower rectum, 4cm from anal verge, invaded to the muscular layer? but didn't penetrate through the serosa. No enlarged lymph nodes at mesorectum, cT2NM0
  2. No synchronous tumor nor polyps at resected colon.
  3. There was no visible liver metastasis, no visible peritoneal seeding

Procedure

- General endotracheal anesthesia was induced and then the patient was placed in modified Trendelenburg lithotomy position. Preoperative antibiotics were given. A Foley catheter and nasogastric tube were placed. The abdomen was prepped and draped in standard sterile fashion. A small vertical incision was made at umbilicus and 12mm trochars were inserted into the peritoneal cavity by open method. Peumoperitoneum was established with CO2 inflation. Laparoscopy was inserted one 12mm trocar was inswerted over RLQ and then another three 5mm trochars were inserted over RUQ, LLQ and LUQ. The operative findings were mentioned as above. The small bowel was inspected and retracted to the right by grasper. The sigmoid colon was then held by grasper and the peritoneum overlying the mesentery was then scored with electrocautery. The inferior mesentery artery was identified, ligated with endoclips, and transected, so was inferior mesentery vein. Dissection was done above the Gerota's fascia and toward the peritoneal attachments. The colon was mobilized from retroperitoneum overlying the sacral promontory and into the pelvis. The arcade vessels to the sigmoid colon were also divided. Sigmoid colon and rectum were mobilized to the level of levator ani. EndoGIC 60-4.1 was used for transect of lower rectum. Midline incision over umbilicus was done, then colon was pulled out. Specimen was resected. cap of EEA 29 was inserted to the colon lumen, then colon was pushed back into abdomen. Pneumoperitoneaum was created again, Apposition of colon was done by EEA 29. One Fr.12 penrose drain was placed at plevis. A transverse incision was made at the RLQ of abdomen,

- right at trochar incision. The terminal ileum was pulled out of the abdomen. A window was created under the ileum and a plastic bridge was placed at this point. The fascia was closed with interrupted sutures of No.1 Vicryl. The skin was closed with 3-0 nylon. The loop ileostomy was then suture with multiple interrupted sutures of 4-0 monoplus placed in such a way as to tack the full thickness of the edge of the terminal ileum to a subcuticular layer of the skin. An ostomy appliance was placed. The patient tolerated the procedure well and was brought to the POR in stable condition.
- 器械數量: 正確
- 紗布數量: 正確

Image

- PAINT
  - colon & rectum

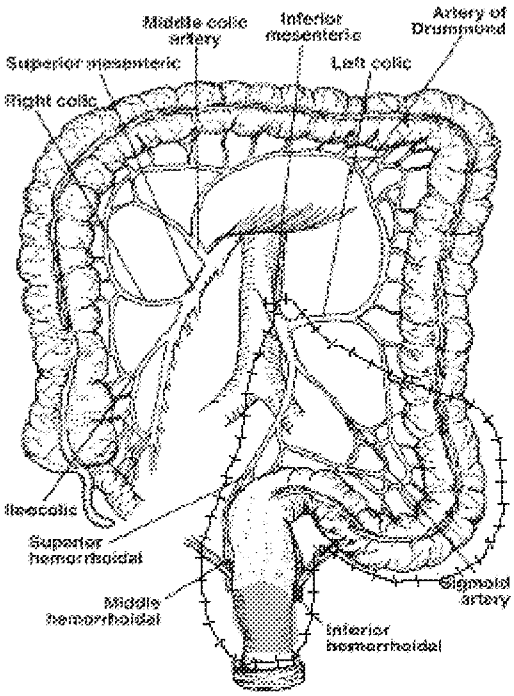

主任 2062F蔣鋒帆 / 醫師 2303K陳映菁

正式摘要(電子簽章：**000000177350978)**

W55 / W55

doctorRank:B
